# A Comparative Review on Enhancing Visual Simultaneous Localization and Mapping with Deep Semantic Segmentation

**DOI:** 10.3390/s24113388

**Published:** 2024-05-24

**Authors:** Xiwen Liu, Yong He, Jue Li, Rui Yan, Xiaoyu Li, Hui Huang

**Affiliations:** 1 Key Laboratory of Urban Land Resources Monitoring and Simulation, Ministry of Natura Resources, Shenzhen 518034, China; 622220900040@mails.cqjtu.edu.cn; 2 School of Smart City, Chongqing Jiaotong University, Chongqing 400074, China; 990020050408@cqjtu.edu.cn (Y.H.); 622230900012@mails.cqjtu.edu.cn (R.Y.); 632201110305@mails.cqjtu.edu.cn (X.L.); 3 College of Traffic & Transportation, Chongqing Jiaotong University, Chongqing 400074, China; 4 Chongqing Digital City Technology Co., Ltd., Chongqing 400074, China; huanghui@dcqtech.com

**Keywords:** semantic segmentation, visual simultaneous localization and mapping, deep learning, dynamic environments, comparative review

## Abstract

Visual simultaneous localization and mapping (VSLAM) enhances the navigation of autonomous agents in unfamiliar environments by progressively constructing maps and estimating poses. However, conventional VSLAM pipelines often exhibited degraded performance in dynamic environments featuring mobile objects. Recent research in deep learning led to notable progress in semantic segmentation, which involves assigning semantic labels to image pixels. The integration of semantic segmentation into VSLAM can effectively differentiate between static and dynamic elements in intricate scenes. This paper provided a comprehensive comparative review on leveraging semantic segmentation to improve major components of VSLAM, including visual odometry, loop closure detection, and environmental mapping. Key principles and methods for both traditional VSLAM and deep semantic segmentation were introduced. This paper presented an overview and comparative analysis of the technical implementations of semantic integration across various modules of the VSLAM pipeline. Furthermore, it examined the features and potential use cases associated with the fusion of VSLAM and semantics. It was found that the existing VSLAM model continued to face challenges related to computational complexity. Promising future research directions were identified, including efficient model design, multimodal fusion, online adaptation, dynamic scene reconstruction, and end-to-end joint optimization. This review shed light on the emerging paradigm of semantic VSLAM and how deep learning-enabled semantic reasoning could unlock new capabilities for autonomous intelligent systems to operate reliably in the real world.

## 1. Introduction

The utilization of visual simultaneous localization and mapping (VSLAM) enabled autonomous agents to operate in unfamiliar environments with greater efficiency. This was achieved through the progressive construction of maps and the estimation of poses. However, the efficacy of traditional VSLAM systems tended to decline in dynamic environments with movable objects. The integration of semantic segmentation into VSLAM enabled the effective differentiation between static and dynamic elements in intricate scenes. The matching and localization in most existing VSLAM systems were dependent on geometric features including points, lines, or planes. These fundamental features often lacked meaningful interpretations of the 3D environment, leading to limitations in distinguishing between static and dynamic elements within the environment.

In recent years, with the development of deep learning, semantic segmentation technologies made significant advancements and found applications in various fields such as autonomous driving, augmented reality, and image editing [1]. Semantic segmentation involved dividing a digital image into multiple segments and assigning each pixel to a specific class, such as person, car, road, sidewalk, vegetation, or building [2]. It offered an efficient method for extracting semantic information from visual data. Many researchers investigated the integration of semantic segmentation into conventional VSLAM systems, showcasing improved performance, particularly in highly dynamic environments. This innovative approach was commonly referred to as semantic VSLAM or semantic SLAM [3]. The semantic segmentation model was primarily utilized in visual odometry, loop closure detection, and environment mapping. Semantic segmentation aided visual odometry in identifying dynamic objects by providing a pixel-level understanding of semantics. Changes in viewpoint, lighting conditions, environmental dynamics, and perceptual aliasing could compromise the flexibility and accuracy of loop closure detection when using original visual features. However, integrating semantic segmentation emphasized stable background structure and provided a powerful clue for enhancing loop closure detection under different conditions. By integrating pixel-level semantic labels, semantic segmentation distinguished various categories such as walls, furniture, objects, and people. This approach explicitly represented the environment rather than just depicting surfaces or geometric primitives, leading to a more comprehensive understanding of the environment and enhancing several aspects. Ultimately, this method improved the intelligent interaction and decision-making ability of environment mapping [4].

However, current research on semantic VSLAM remained constrained in its scope and scale. There was a notable absence of a systematic review and comparative analysis within this evolving domain. The majority of published studies concentrated on isolated algorithm development and experimental verifications using limited datasets. The interrelations between semantic segmentation and various aspects of VSLAM were not exhaustively explored. Furthermore, a comprehensive performance benchmark and evaluation across diverse solutions were lacking. Therefore, it is important to undertake a review of the cutting-edge semantic segmentation technologies implemented in VSLAM to illuminate potential avenues for future research.

**Figure 1:**
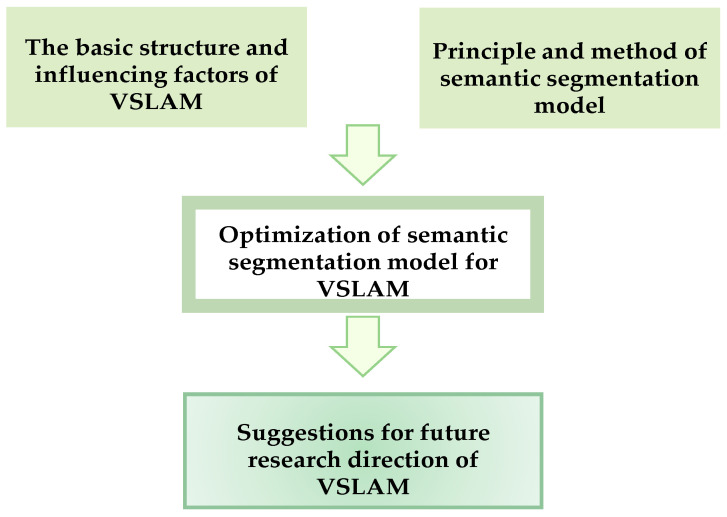
General discussion framework.

The main objective of this study was to present a comprehensive overview of the development and applications of VSLAM. It summarized and compared the various applications of semantic segmentation in the key components of VSLAM, outlining their roles, benefits, and limitations. Additionally, research gaps in the current literature were identified, and promising future directions in this field were highlighted. The subsequent sections of this paper are structured as follows: Section 2 introduces the fundamental technologies and influential factors in the workflow of traditional VSLAM systems. Section 3 outlines the general principles and classification of mainstream semantic segmentation approaches, with a particular focus on deep learning-based methods. In Section 4, diverse applications of semantic segmentation in the key components of VSLAM are elaborated upon. Finally, Section 5 concludes the paper by summarizing the key findings and discussing potential future research directions. The architecture for this paper is shown in Figure 1.

## 2. Key Technologies and Influencing Factors of VSLAM

### 2.1. Workflow of VSLAM

**Figure 2:**
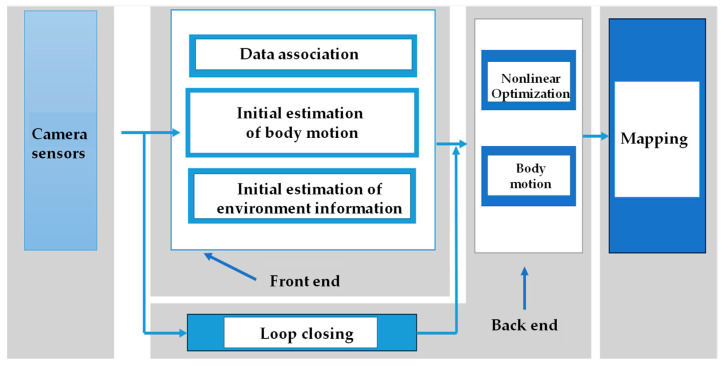
A basic framework for simultaneous localization and mapping.

The technologies of VSLAM were categorized into four primary components: front-end visual odometry, back-end optimization, loop closure detection, and mapping. The front-end visual odometry involved extracting features from sequences of images and matching them between frames to calculate the incremental motion of the camera pose, enabling real-time localization but being susceptible to drift accumulation over time [5]. The back-end optimization refined the poses by minimizing the discrepancies between predicted and observed feature locations within a temporal window, thereby mitigating accumulated drift [6]. Loop closure detection identified previously visited locations upon revisiting them, establishing constraints between current and past poses to constrain drift. The mapping module combined visual data and optimized poses to progressively construct a map of unfamiliar environments [7]. The typical workflow and interconnection among these modules are illustrated in Figure 2.

### 2.2. Front-End Visual Odometry

Two primary approaches were employed for visual odometry: feature-based methods, which involved matching sparse features across frames, and direct methods, which analyzed the intensities of all pixels. Feature-based methods, including ORB-SLAM [8], were renowned for their robustness but could encounter difficulties in textureless regions. In contrast, direct methods, exemplified by LSD-SLAM [9], bypassed explicit data association but exhibited lower resilience in dynamic scenes. Semi-direct methods aimed to strike a balance between the two by tracking sparse features and minimizing photometric errors. Challenges encountered in visual odometry included illumination variations, motion blur, occlusions, and the presence of dynamic objects in the surroundings.

Numerous approaches have been put forward to enhance the robustness of visual odometry. Optimization of feature extraction algorithms including scale-invariant feature transform (SIFT) and oriented FAST and rotated BRIEF (ORB) was pursued, and the integration of visual data with inertial measurements had demonstrated improved accuracy in challenging scenarios. For instance, Ortiz, et al. [10] integrated SIFT into position- and scale-invariant feature transform (PSIFT), refining SIFT to 48 bytes, with PSIFT exhibiting comparable performance to SIFT while achieving enhanced accuracy and efficiency, outperforming most contemporary binary descriptors. Additionally, Wang, et al. [11] combined self-motion estimation with sequence-based learning using deep neural networks. Specifically, they utilized Convolutional Neural Networks (CNNs) to estimate camera motion in optical flow and modeled motion dynamics using Recurrent Neural Networks (RNNs). However, in complex or dynamic environments, traditional front-end visual odometry positioning was often not accurate enough, resulting in an escalating error over time.

### 2.3. Back-End Optimization

Two primary categories of approaches were employed for back-end optimization: filtering methods and smoothing methods. Filtering methods, including the Extended Kalman Filter (EKF), iteratively updated pose estimates by incorporating motion dynamics and observations [12]. On the other hand, smoothing methods, like bundle adjustment, optimized poses within a sliding window by minimizing the reprojection errors of all features. While filtering methods were computationally efficient, smoothing methods traded off increased accuracy for higher computational costs.

In addition to standard optimization techniques, various enhancements were introduced, including the integration of geometric constraints and the utilization of geometric constraint-based joint optical flow for identifying dynamic feature points. Zhao and Vela [13] introduced the maximum logarithm of determinant (Max-logDet) metric to guide feature selection in least-squares pose optimization, demonstrating through experiments that optimized least-squares algorithms could achieve effective feature selection, thereby significantly improving pose tracking accuracy. Furthermore, Zhao and Vela [14] proposed a method that extracted the most informative segments from each 3D line through appropriate line segment segmentation for pose optimization formulations. The results illustrated that precise line segmentation could enhance pose estimation accuracy, although the limitation lay in the incapacity of low-level geometric features to semantically handle dynamic environments. In environments that lacked texture, traditional loop closure detection methods might have struggled to function effectively and could have been sensitive to variations in lighting, potentially resulting in erroneous or overlooked detections.

### 2.4. Loop Closure Detection

Loop closure detection served to determine whether the camera revisited a previously mapped region, offering constraints to minimize drift [15]. The main approaches included appearance-based methods utilizing image retrieval techniques and learning-based methods that leveraged CNNs.

Appearance-based methods commonly utilized the bag-of-words model to create global image descriptors. Several studies enhanced VSLAM algorithms by refining the bag-of-words model [16]. Shen, et al. [17] proposed a loop closure detection algorithm based on an enhanced real-time updating bag-of-words model. By extracting feature descriptors from online images and integrating them with preloaded offline words, a customized bag-of-words specific to the mobile robot’s operational environment was generated. This tailored bag-of-words adapted to the robot’s specific application scene, thereby enhancing the system’s resilience. Additionally, Xi, et al. [18] proposed a slam-dunk loop closure detection algorithm that optimized the bag-of-words model. They enhanced the clustering algorithm to create the offline vocabulary tree and utilized an improved K-Means algorithm for vocabulary tree construction. Furthermore, they continually updated the vocabulary tree with image feature data from the real-world scene to enhance its representational capabilities. Although these approaches were successful in location recognition, they heavily depended on viewpoint invariance and robust feature extraction [19].

### 2.5. Mapping

Based on different front-end visual odometry methods, the resulting maps were categorized into sparse, semi-dense, and dense maps [20]. Sparse maps, generated from feature-based odometry, consisted of point landmarks. Semi-dense maps incorporated certain surface elements computed through direct methods. Dense maps estimated depths for all pixels using stereo or monocular depth estimation techniques. While denser maps offered more detailed information about structures and textures, they required more intensive computational resources.

The quality of map construction in VSLAM was heavily influenced by environmental features. In environments with rich textures and distinctive features, VSLAM algorithms typically excelled in map building and localization estimation. Conversely, performance might have deteriorated in environments with limited textures or sparse features. The conventional ORB-SLAM was a feature-based approach that constructed sparse maps from point clouds but might have lacked the necessary detail to accurately identify specific objects [8]. Consequently, Sunderhauf et al. [21] introduced an object-oriented semantic mapping method that integrated a single-shot multi-box detector (SSD) and ORB-SLAM2. This approach dynamically maintained and updated point clouds for each object class; however, the individual management and updating of each object in the map presented challenges for robots in distinguishing objects of the same category in practical scenarios [22].

Metrics used to assess mapping accuracy encompassed absolute trajectory error for global consistency, relative pose error for drift assessment, and completeness to gauge the extent of the true environment coverage [23]. The fidelity of reconstructed maps was significantly influenced by the richness of features present in the perceptual environments.

### 2.6. Evaluation Metrics

Various metrics were proposed to assess and compare the performance of different VSLAM systems. The most frequently utilized metrics included absolute trajectory error (ATE), relative pose error (RPE), and map completeness [24].

#### 2.6.1. Absolute Trajectory Error (ATE)

The *ATE* metric quantified the global consistency between the estimated trajectory and the ground truth trajectory. It was computed as the root-mean-squared error (RMSE) between the positions of estimated poses and ground truth poses at each timestamp.

(1) $$ATE=\sqrt{\frac{1}{N}\sum_{i=1}^{N}{\left||\mathrm{trans}(T_{\mathrm{gt},i}^{-1}T_{\mathrm{est},i})|\right|}_{2}^{2}}$$

where est,*i* and gt,*i* are the positions of estimated and ground truth poses at time *i*, and *N* is the total number of poses. *ATE* evaluates the overall localization accuracy of the VSLAM system. Lower *ATE* indicates better global consistency. However, *ATE* does not reflect the drift or stability of the system.

#### 2.6.2. Relative Pose Error (RPE)

*RPE* quantified the error in the relative pose changes between two time steps over a fixed time interval Δ*t*:

(2) $$RPE_{\mathrm{trans}}=\sqrt{\frac{1}{N-\Delta t}\sum_{i=1}^{N-\Delta t}{\left||\mathrm{trans}{\left((T_{\mathrm{gt},i}^{-1}T_{\mathrm{gt},i+\Delta t})\right)}^{-1}{\left(T_{\mathrm{esti},i}^{-1}T_{\mathrm{esti},i+\Delta t}\right)}^{\vee }|\right|}_{2}^{2}}$$

where trans represents the translation part of the variable inside the parentheses; esti,*i* and gt*,i* are the positions of estimated and ground truth poses at time *I*; ∆*t* indicates the interval time; and *N* is the number of relative pose changes being compared [25]. RPE effectively evaluates the local drift of VSLAM algorithms over a given time interval. Lower RPE indicates more stable odometry output and smaller drift. But *RPE* does not quantify global consistency or absolute accuracy.

#### 2.6.3. Map Completeness

In addition to localization accuracy, the completeness of reconstructed maps was another crucial metric, particularly for robot navigation and planning [26]. More comprehensive maps offered greater detail about objects, structures, and textures within the environments. However, quantifying map completeness was more challenging compared to numerical pose errors. Currently, some researchers examined the integrity evaluation of maps based on volume, surface, and semantic classification, as outlined in Table 1.

While completeness metrics based on volumetric, surface, or semantic classifications have been proposed, standardized benchmarks are still lacking.

In summary, ATE and RPE were the two most widely adopted metrics for evaluating VSLAM systems in terms of localization accuracy and drift. Map completeness was also significant but lacked standardized quantitative benchmarks. Employing these complementary metrics provided a more comprehensive understanding of the performance of VSLAM algorithms and systems.

### 2.7. Influencing Factors

The performance of VSLAM systems could be influenced by numerous factors pertaining to the environments, sensors, algorithms, and system capabilities. These factors determined the accuracy, robustness, and efficiency with which a VSLAM system could operate in real-world conditions. The primary influencing factors are summarized below:

Environments: The perceptual environments imposed fundamental constraints on the quality of visual observations and features that could be extracted [27], directly impacting the performance of VSLAM algorithms. Environments characterized by rich textures and stable lighting conditions were more conducive to optimal performance, whereas low-textured areas, repetitive patterns, reflective surfaces, and varying illuminations could diminish visual processing and data association capabilities. Highly dynamic environments with numerous moving objects could also adversely affect motion estimation and map construction [28]. Field environments typically present more challenges compared to indoor settings. A consistent solution to mitigate the interference caused by dynamic objects in SLAM systems involves employing object detection and image segmentation algorithms to filter out dynamic regions in the images before visual odometry. Subsequently, the camera’s approximate position is computed using static environmental points, and a map containing semantic information is generated.

Sensing modalities: The types and characteristics of visual sensors determined the visual information perceived by the VSLAM system [29]. Monocular cameras imposed fewer constraints on motion estimates compared to stereo or red, green, blue, and depth (RGB-D) cameras. Range sensors including lasers and depth cameras provided geometric structures more directly but lacked color and texture details. Parameters including field of view, resolution, frame rate, exposure time, and other intrinsic factors also influenced the amount of usable information captured from the surroundings. Multi-sensor fusion involving inertial, global positioning system (GPS), or other exteroceptive data could potentially compensate for the limitations of individual sensors. The model of Frustrum PointNets integrated both RGB cameras and light detection and ranging (LiDAR) sensors to enhance scene understanding accuracy [30].

The feature extraction, data association, motion estimation, and map optimization components within the VSLAM system collectively contributed to its overall performance, robustness, and efficiency. Utilizing more repeatable feature detectors and descriptors enhanced data association for tracking and loop closure. The choice between filtering and smoothing techniques introduced a trade-off between efficiency and accuracy. Moreover, map representations impacted interpretability, storage requirements, and computational complexity [31]. The development of you only look at coefficients with dynamic convolutions (YOLACT-Dyna) aimed to eliminate potential moving objects in the scene and provide an approximate camera pose estimation. Subsequently, leveraging the camera pose and polarity constraints, the algorithm calculated motion probabilities for each potential moving object. Finally, motion feature points were filtered out, and the pose was computed using static feature points.

Existence of loops: Detected loops presented crucial opportunities to diminish accumulated drift and strengthen global map consistency. Smaller loops with shorter intervals aided in constraining pose errors more frequently [32]. However, repetitive environments with ambiguous appearance information could result in false loop detections. The quantity, size, and frequency of loops in the trajectory fundamentally influenced performance [33].

Motion dynamics: Highly dynamic movements characterized by high speed, sudden rotations, and aggressiveness could have a detrimental impact on visual processing in VSLAM. Motion blur and rolling shutter effects could degrade visual feature extraction and matching [34]. Moreover, complex maneuvers further complicated motion estimation, potentially violating the assumptions of typical VSLAM algorithms. Wen, et al. [35] introduced a novel visual SLAM approach named DP-SLAM, which relied on sparse feature tracking and integrated the concept of motion probability. A propagation model was utilized for dynamic keypoint identification. This methodology was integrated into the front end of the ORB-SLAM2 system, serving as a preprocessing stage to filter out keypoints associated with moving objects. Furthermore, the backgrounds of frames occluded by identified dynamic objects were painted over, offering benefits for applications including virtual reality and augmented reality.

**Figure 3:**
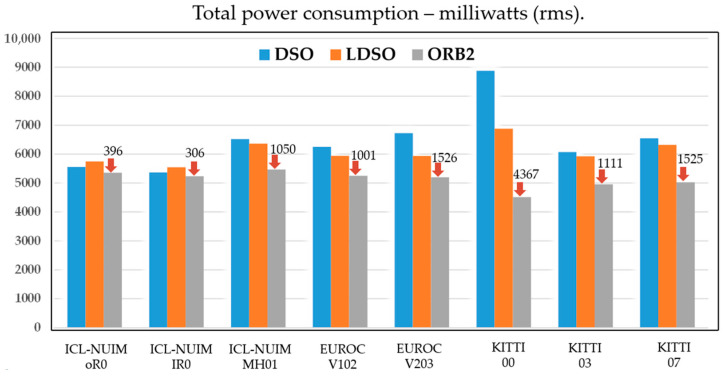
Comparison of energy consumption between classic simultaneous localization and mapping frameworks [37].

Hardware capabilities: The computational capacity of onboard processors and co-processors determined the feasibility of deploying computationally intensive algorithms for real-time VSLAM. Additionally, power consumption and heat dissipation presented constraints on embedded system design [36]. The available onboard memories limited the duration and resolution of mapping sessions, while communication bandwidth impacted multi-robot collaborative mapping capabilities. YANIK, et al. [37] conducted an evaluation and comparison of three visual SLAM methods—ORB-SLAM2, direct sparse odometry (DSO), and DSO with loop closure (LDSO)—in terms of energy consumption and resource usage, as shown in Figure 3.

In summary, a multitude of interrelated factors encompassing environments, sensors, algorithms, hardware, loops, and motion impacted the accuracy, robustness, and efficiency of VSLAM systems. A comprehensive understanding of these influential factors offered valuable insights into the trade-offs and constraints associated with various design decisions. This understanding propelled research efforts towards the development of more comprehensive and resilient solutions for VSLAM in challenging real-world scenarios. 

## 3. Principles and Methods of Semantic Segmentation

Semantic segmentation involved assigning semantic labels including person, car, road, etc., to each pixel in an image, offering a powerful method to extract high-level understanding from visual data. With the resurgence of deep learning in recent years, CNNs emerged as the predominant approach, demonstrating remarkable success in semantic segmentation [38]. This section begins by presenting the definition and evaluation metrics of semantic segmentation. Subsequently, common network architectures are discussed, with a focus on encoder–decoder-based frameworks. Finally, factors influencing the performance of semantic segmentation models are summarized.

### 3.1. Definition and Evaluation Metrics

Semantic segmentation aimed to divide an image into non-overlapping regions with the same semantics, essentially framing it as a pixel-level classification challenge. Given an input image (*I*) comprising *N* pixels, semantic segmentation assigned a semantic label (*l_i_*) to each pixel (*i*), with *l_i_* drawn from a predefined label set (*L*) containing *K* potential classes. The output was a label map with dimensions matching those of the input image.

Two commonly used evaluation metrics for semantic segmentation were pixel accuracy and mean intersection over union (mIoU) [39]. Pixel accuracy quantified the percentage of correctly classified pixels, while mIoU assessed the overlap between predicted and ground truth masks for each class before averaging the results. Higher values indicated superior segmentation performance. Additional metrics like frequency-weighted intersection over union (IoU) could accentuate the importance of rare classes.

### 3.2. Encoder–Decoder Architectures

Most state-of-the-art semantic segmentation models leveraged convolutional encoder–decoder architectures [40]. The encoder progressively reduced spatial resolution and extracted visual features through a sequence of convolutional and pooling layers. Conversely, the decoder symmetrically restored object details and spatial dimensions via upsampling and convolutions, enabling the integration of semantic insights from deep layers with fine-grained details from earlier layers.

Earlier designs converted CNN classifiers into fully convolutional networks (FCNs) and introduced upsampling layers to recover spatial intricacies [41]. U-Net innovatively introduced skip connections linking corresponding encoder and decoder layers to incorporate multi-scale semantic information [42]. Numerous researchers worldwide refined semantic segmentation models tailored to diverse scenarios. The semantic segmentation network (SegNet) model, proposed by Badrinarayanan et al. [43], stood out as a representative encoder–decoder algorithm for road and vehicle segmentation. Compared to the architecture of FCNs, SegNet boasted a significantly reduced size. This reduction was primarily attributed to the utilization of positional information in SegNet from recorded pooling operations instead of direct deconvolution processes. In SegNet, the pooling layers not only retained maximum values but also stored the spatial positions of these maxima in the original image. This approach facilitated accurate mapping of relevant values to their respective positions during upsampling, thereby enhancing the accuracy of the reconstructed image.

Some researchers also focused on optimizing models from a structural standpoint. For instance, the refinement network (RefineNet) employed a multi-path refinement architecture to fuse features from various levels, and DeepLabv3 enhanced the encoder with atrous convolutions to encode multi-scale context [44]. The pyramid scene parsing network (PSPNet) integrated a pyramid pooling module before the decoder to aggregate contextual information [45]. In recent years, new models were proposed to address the degradation problem in order to achieve higher accuracy with a large number of convolutional layers. While some of these models demonstrated improved results [46], achieving an appropriate trade-off between efficiency and accuracy remained a challenging task for large datasets. Kazerouni et al. [47] proposed the Ghost-Unet model as an asymmetric encoder–decoder structure for high-precision semantic segmentation, considering a reasonable number of convolutional layers, which received positive feedback in terms of accuracy and efficiency.

### 3.3. Context Modeling Modules

To incorporate broader contextual information beyond local receptive fields, various context modeling modules were introduced. Atrous Spatial Pyramid Pooling (ASPP) scanned an image with filters at multiple sampling rates to capture multi-scale information [48]. Non-local networks utilized non-local operations to capture long-range dependencies. Algorithms that integrated contextual information aimed to enhance the accuracy and robustness of semantic segmentation by leveraging local and global information, as well as features from different scales. These algorithms were better equipped to address challenges including complex scenes and unclear boundaries, leading to improved semantic segmentation outcomes. Noteworthy algorithms included conditional random fields (CRFs) [49], dilated convolutions [50], and multi-scale predictions [51].

CRFs further refined predictions by smoothing using pixel affinities. Attention mechanisms dynamically aggregated contextual information surrounding regions of interest. These modules enhanced contextual representations and relationships to improve segmentation accuracy. The seminal model for conditional random fields was the DeepLabv1 model, introduced by CHEN et al. [52]. It incorporated a fully connected CRF model as an independent back-end processing step to optimize segmentation results. Each pixel was treated as a node within the region, and the association between two pixels, regardless of distance, influenced pixel label classification. This strategy aided in recovering local details that may be lost due to the spatial invariance of CNNs. Although the fully connected model was computationally intensive, the DeepLabv1 model employed approximate algorithms to significantly reduce computational costs. However, these methods overlooked the specificity of class weights in the classification layer. Zhu et al. [29] observed that class weights for neighboring boundary pixels often lacked discrimination, thereby hindering performance. To address this issue, a novel approach called embedded conditional random fields (E-CRF) was proposed. E-CRF seamlessly integrated CRFs into the CNNs to achieve more efficient end-to-end optimization. It employed CRFs to facilitate message passing between pixels in high-level features and refined the feature representation of boundary pixels by utilizing internal pixels belonging to the same object.

Dilated convolutions, also known as atrous convolutions, were initially introduced as a signal processing technique. In CNNs, dilated convolutions could significantly expand the receptive field without introducing additional parameters. Consequently, large-scale pooling operations were unnecessary to enlarge the receptive field, preventing the loss of fine-grained information associated with pooling. Dilated convolutions were often coupled with multi-scale predictions. Zheng, et al. [53] established a spatial pyramid (ASPP) by connecting multiple dilated convolutions in parallel. ASPP employed dilated convolutions with varying dilation rates to conduct diverse convolution operations on the feature map. It achieved a larger receptive field than the size of its convolution kernel without increasing the number of parameters. Notably, the size of the feature map remained unchanged post dilated convolution. However, dilated convolutions encountered the gridding issue, where zeros were inserted between two sampled pixels of the convolution kernel. Excessive dilation rates could lead to overly sparse convolutions, resulting in inadequate information capture due to sparse input sampling and hindering effective model learning.

The core principle behind multi-scale predictions was to expand the receptive field across multiple resolutions and effectively enrich feature information of the target task by merging features from various scales, thereby enhancing segmentation accuracy. Integrating multi-scale predictions with CRFs could further enhance segmentation accuracy. This network effectively harnessed both local and global contextual information, spanning from the entire scene to each pixel, to carry out pixel-level label estimation. For example, Ding et al. [53,54] proposed a segmentation network called CGBNet, which improved segmentation performance through context encoding and multipath decoding. This network first generated local features compared with context through a context coding module to make use of informative context and discriminating local information. This context-coded module greatly improved segmentation performance, especially for objects that were not obvious.

### 3.4. Training Strategies

In many instances, manual annotation was required for semantic segmentation tasks as training data. Semantic segmentation models were designed to conduct pixel-level image segmentation [55], where each pixel was assigned a semantic label to delineate the boundaries and categories of various objects and regions within the image. This typically necessitated detailed pixel-level annotations that specified the semantic category to which each pixel belonged. To alleviate the demand for manual annotation, weakly supervised semantic segmentation (WSSS) [56] based on image-level labels garnered attention due to its reduced annotation cost.

Existing methods often leveraged class activation maps (CAMs) to assess the correlation between image pixels and classifier weights. However, classifiers tended to focus solely on discriminative regions, disregarding other valuable information in each image, leading to incomplete localization maps. To tackle this challenge, Chen et al. [3] introduced a self-supervised methodology known as Self-supervised image-specific prototype exploration (SIPE), comprising image-specific prototype exploration (IPE) and general specific consistency (GSC) losses. Specifically, IPE tailored prototypes for each image to capture comprehensive regions, forming image-specific CAMs (IS-CAMs). GSC was implemented to ensure alignment between the general CAMs and the specific IS-CAMs, thereby refining feature representation and strengthening the self-correcting capability of prototype exploration. Additionally, the model benefited from data augmentation techniques including scaling, cropping, flipping, and rotation to prevent overfitting and enhance robustness.

Liu, et al. [57] proposed a novel concept termed projection onto orthogonal proto-types (POP), which updated features to recognize new classes without impacting the base classes. A collection of orthogonal prototypes was established in POP, with each prototype representing a semantic class, and each class was predicted by the features projected onto its respective prototype. Uniform class sampling was employed to ensure equal contribution from each class during training [58]. Online hard example mining focused on instances that were misclassified, thereby enhancing performance in challenging scenarios. Joint training on multiple datasets with diverse data distributions bolstered the model’s generalization capability.

Nevertheless, traditional distillation methods had struggled with LiDAR-based semantic segmentation due to the complexities posed by the sparsity, randomness, and density variations of point clouds. Hou, et al. [59] proposed the utilization of output distillation for point-wise and voxel-wise information to complement sparse supervision signals. The complete point cloud was partitioned into multiple super-voxels, and a difficulty-aware sampling strategy was devised to more frequently sample super-voxels containing low-frequency classes and distant objects. Point-wise and voxel-wise affinity distillation was implemented on these super-voxels, leveraging similarity information between points and super-voxels to aid the model in capturing structural details about the surrounding environment more effectively. In order to address the issue of data imbalance in semantic segmentation, Zhu et al. [60] proposed a central sampling strategy to evenly select training samples from each class in each epoch. A rapid training program was also introduced to reduce the computational burden. This allowed the researchers to explore the use of a large number of false labels. The network structure of data-adaptive transformer (DAFormer) proposed by Hoyer et al. [58] consisted of a Transformer encoder and a multi-level context-aware feature fusion decoder. It represented a significant advancement for unsupervised domain adaptation.

### 3.5. Factors Affecting Performance

The performance of semantic segmentation models was influenced by a variety of factors including network architecture, training data, optimization strategies, model regularization, contextual modeling, and hardware resources. The intricate interplay of these factors ultimately dictated the accuracy, speed, and robustness of the models.

Network Architecture Design: The overall capacity, depth, receptive field size, and path aggregation strategies within network architectures significantly impacted feature learning and the capabilities for multi-scale contextual modeling. Larger and deeper backbones including ResNet [61] facilitated the encoding of more robust features. Expanding receptive fields through atrous convolutions or spatial pyramid pooling enabled the capture of broader context. Advanced decoder modules featuring skip connections facilitated the fusion of hierarchical features from varying scales. Many existing methods compromised spatial resolution to achieve real-time inference speed, resulting in diminished performance. Yu, et al. [62] devised a small stride spatial path to preserve spatial details and generate high-resolution features. Concurrently, they employed a context path with a rapid downsampling approach to acquire ample receptive field coverage. Introducing a new feature fusion module on top of these two paths effectively amalgamated features, leading to an mIoU of 68.4% on the Cityscapes test dataset [63].

Training Data: The quantity, quality, and diversity of annotated training data fundamentally determined the performance boundaries of models. Larger datasets with diverse annotation variations contributed to enhanced generalization. High-quality labels featuring precise segmentation boundaries enabled models to grasp finer details. Class balancing [64] and data augmentation [65] further bolstered robustness. Kenjic, et al. [66] introduced outlier removal for re-labeling, class-driven balancing of validation and training datasets, and targeted image processing for underrepresented classes. Evaluation outcomes demonstrated enhanced inference accuracy compared to utilizing common open-source datasets, achieving an mIoU of 68.4%.

Loss Functions: The selection of pixel-wise loss functions influenced model training behavior [67]. Weighted loss functions addressed class imbalance challenges, while bootstrapped loss focused on challenging examples. Lovász loss directly optimized the IoU metric. Combining losses with distinct focuses contributed to overall performance improvement. The choice of loss function typically hinged on the characteristics of the training dataset, including its distribution, skewness, and boundaries. Focal-based loss functions were beneficial for highly imbalanced segmentation tasks. Binary cross-entropy was suitable for balanced datasets, whereas mildly skewed datasets could benefit from smooth or generalized dice coefficient.

Optimization Schemes: Optimization hyperparameters including batch size, learning rates, and schedules played a pivotal role in model convergence and training efficiency [68]. Larger batch sizes leveraged batch normalization but necessitated increased GPU memory. Well-calibrated learning rate schedules and warm-up strategies expedited convergence. The online retraining approach empowered the segmentation network to effectively learn from confident regions biased towards accurate labels.

Context Modeling: Multi-scale context aggregation modules heightened localization and recognition capabilities by capturing broader contextual information. Atrous convolutions, pyramid pooling, and non-local operations offered complementary contextual representations [69]. Conditional random fields further refined predictions based on pixel affinities. Li, et al. [70] proposed a novel context-based cascaded network, CTNet, which delved into spatial and channel contextual information to unveil semantic contexts for semantic segmentation. The spatial context module leveraged pixel-class correlations to unveil spatial contextual dependencies among pixels [71]. Simultaneously, the channel context module modeled long-term semantic relationships between channels to learn semantic features encompassing semantic feature maps and class-specific features. By utilizing the acquired semantic features as prior knowledge to guide network learning, CTNet captured more precise long-range spatial dependencies.

Regularization: Various regularization techniques were employed to mitigate overfitting to the training data [72]. Weight decay restricted weight norms, while data augmentation techniques like flipping, scaling, cropping, and elastic deformations enhanced training sample diversity. Dropout randomly deactivated units during training to prevent co-adaptation. Yuan, et al. [73] introduced a novel form of batch normalization known as distribution-specific batch normalization (DSBN) to address this issue. They underscored the significance of robust augmentation methods for semantic segmentation and achieved state-of-the-art outcomes in the semi-supervised context using urban landscape and Pascal VOC datasets.

Hardware Resources: Dedicated GPUs and TPUs expedited training and inference by efficiently parallelizing operations. Swifter hardware facilitated training larger models, handling bigger batches, and extending training schedules. Model optimization through pruning, quantization, and distillation tailored intricate models for deployment [74].

### 3.6. Representative Models

While earlier studies utilized FCN-based models including DeconvNet [75] and SegNet, which offered limited context modeling capabilities, recent approaches have adopted more sophisticated models like Mask R-CNN [76], DeepLabv 3+ [77], and PSPNet [45]. These advanced models provided enhanced multi-scale contextual reasoning, resulting in more precise segmentations. Additionally, lightweight models like ERFNet [78] were employed for efficient inference on embedded devices. The selection of a semantic segmentation model directly influenced the quality of semantic information. Table 2 lists the prevailing semantic segmentation models in the field.

When selecting a semantic segmentation model, the initial consideration was the choice of network architecture. Different network architectures, including CNNs, RNNs, or variational autoencoders (VAEs), offered distinct advantages for various semantic segmentation tasks. For instance, CNNs excelled in processing data with spatial continuity, including images and videos, while RNNs were more adept at handling data with temporal continuity, like speech and text. Secondly, the curation and generation of datasets played a crucial role. A diverse and realistic dataset could enhance the model’s generalization capability, enabling it to make accurate predictions even with unseen data. Moreover, judicious data augmentation strategies, including rotation, scaling, cropping, etc., could assist the model in adapting to diverse scenarios and enhancing its robustness. During the model training phase, the choice of regularization method was equally vital. Techniques like L1 and L2 regularization could manage the model’s complexity and prevent overfitting, where the model performed well on training data but poorly on test data. Additionally, methods like Early Stopping could be employed to mitigate overfitting. The selection of hardware resources also impacted the model training speed. Leveraging high-performance GPUs for model training could significantly accelerate the training process, thereby enhancing the model’s iteration speed. Lastly, advancements in neural network architectures, training methodologies, and accelerated computing offered substantial support for the advancement of semantic segmentation models. In the future, researchers could leverage deep learning and artificial intelligence techniques to drive further progress in the development of semantic segmentation model.

## 4. Applications of Semantic Segmentation in VSLAM

Traditional VSLAM systems traditionally relied on low-level geometric features for localization and mapping [81]. In contrast, semantic segmentation offered a high-level understanding of environments by assigning semantic labels to image pixels. This capability enabled the differentiation between static and dynamic components in complex scenes. By integrating semantic segmentation, the accuracy and robustness of VSLAM could be enhanced in highly dynamic real-world environments [82]. In this section, various applications of semantic segmentation in key components of the VSLAM pipeline are outlined. Subsequently, a comparative analysis of representative semantic segmentation models utilized in cutting-edge VSLAM systems is conducted.

### 4.1. Visual Odometry

**Figure 4:**
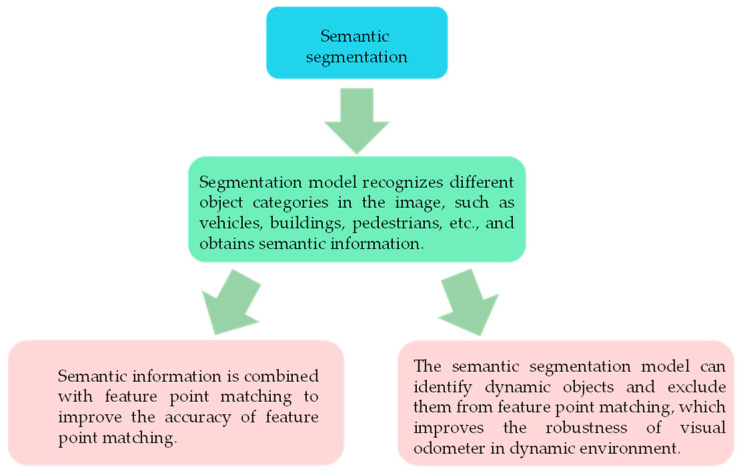
Optimization process of visual odometer based on semantic segmentation.

Visual odometry played a pivotal role in the VSLAM pipeline, aiming to estimate the incremental camera motion between consecutive frames. Traditional visual odometry relied on tracking and matching low-level geometric features, including points, lines, or keypoints, across frames to compute changes in camera pose [83]. However, these low-level features lacked semantic understanding of the 3D environment, hindering their ability to distinguish between static and dynamic elements in complex environments encompassing both background structures and independently moving objects. Figure 4 illustrates the optimization process of semantic segmentation for visual odometry. Features originating from moving objects could compromise the accuracy and robustness of visual odometry.

**Figure 5:**
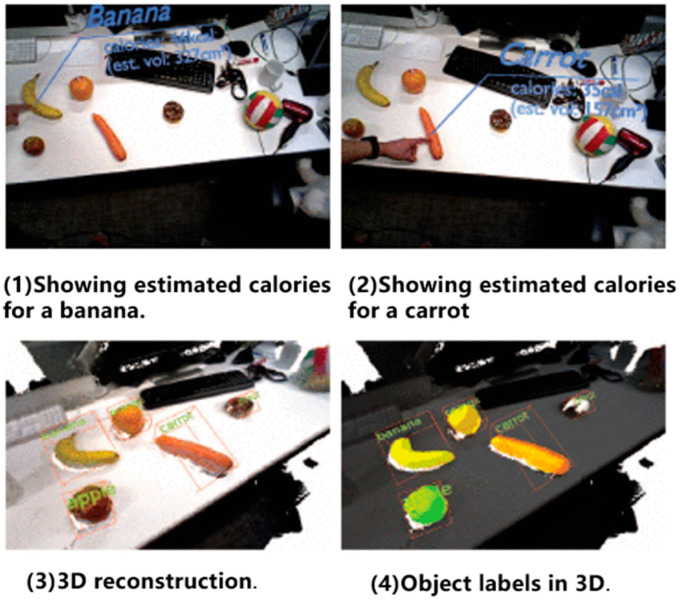
The model succeeded in segmenting the fruit [84].

Semantic segmentation offered a viable solution to overcome this limitation by providing pixel-level semantic comprehension to recognize dynamic objects. The segmented masks could be utilized to filter out features related to moving objects during the estimation of ego-motion between frames, consequently enhancing odometry accuracy in dynamic environments. Notable works that integrated semantic segmentation for robust visual odometry included:

MaskFusion [84]: This approach utilized the CNNs to segment RGB images into static scenes and moving objects, subsequently eliminating masks corresponding to moving objects during feature tracking and matching across frames. This methodology significantly enhanced the efficacy of visual odometry in dynamic environments by mitigating the impact of dynamic objects on camera pose estimation. Such an approach could effectively boost the performance of visual odometry, particularly in intricate environments characterized by numerous dynamic objects. Figure 5 illustrates an example of a real-life scenario.

**Figure 6:**
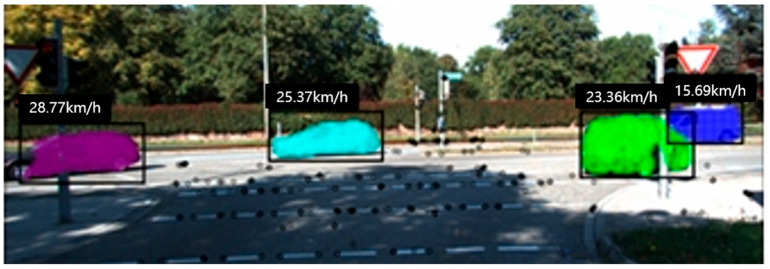
VDO-SLAM can track vehicles and estimate their speeds [85].

VDO-SLAM [85]: Utilizing Mask R-CNN for instance segmentation and object tracking, this method excluded objects exhibiting independent motion from ego-motion estimation between frames. VDO-SLAM integrated optical flow or alternative object tracking techniques to monitor objects across consecutive frames. This strategy facilitated the tracking of dynamic objects during camera pose estimation, thereby enhancing the efficacy of visual odometry in dynamic environments. This approach effectively elevated the performance of visual odometry, particularly in intricate environments with a significant presence of dynamic objects. Figure 6 shows the dynamic tracking and recognition of vehicles.

ORB-SLAM3 [86]: ORB-SLAM3, a widely used open-source VSLAM system, incorporated semantic segmentation functionalities into its framework. This system was capable of identifying and tracking semantic objects within the environment, thereby bolstering the stability and scene comprehension capabilities of SLAM. Leveraging the outcomes of semantic segmentation, ORB-SLAM3 could detect and track dynamic objects in the scene. Subsequently, this technique was employed for dynamic object filtering, enabling the filter to mitigate the impact of dynamic objects, thereby enhancing the stability of visual odometry.

SLAM-Net [87]: SLAM-Net was a deep learning model that integrated visual SLAM with semantic segmentation, offering applications in intelligent navigation, autonomous driving, and robotics to enhance environmental perception and path planning capabilities. Through predominantly employing end-to-end training techniques, SLAM-Net enhanced visual odometry. In specific indoor settings, SLAM-Net demonstrated superior performance compared to conventional learned visual odometry methods.

Experiments conducted using these approaches showcased improved accuracy and robustness in contrast to conventional geometry-based visual odometry, particularly evident in highly dynamic outdoor driving datasets. Semantic segmentation offered crucial perception capabilities to segregate complicating elements from odometry estimation in intricate scenes. This breakthrough unleashed new possibilities for VSLAM to function dependably in real-world scenarios [88].

### 4.2. Loop Closure Detection

**Figure 7:**
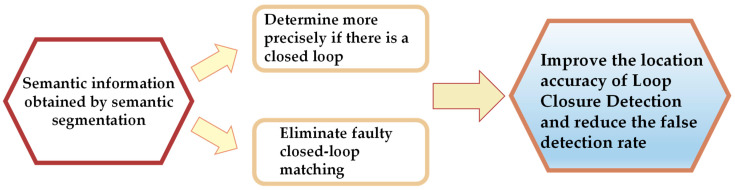
Semantic segmentation to improve the loop closure detection process.

Loop closure detection was a crucial component in VSLAM systems designed to address drift and enhance global consistency. It involved identifying situations where the camera revisited a previously visited location and establishing pose constraints between the current and previous positions. Conventional methods relied on image retrieval and feature matching guided by visual appearance cues [89,90]. However, variations in viewpoint, lighting conditions, environmental dynamics, and perceptual aliasing could undermine the robustness and accuracy of loop closure detection using raw visual features. Figure 7 illustrates the process of enhancing loop closure detection through semantic segmentation.

**Figure 8:**
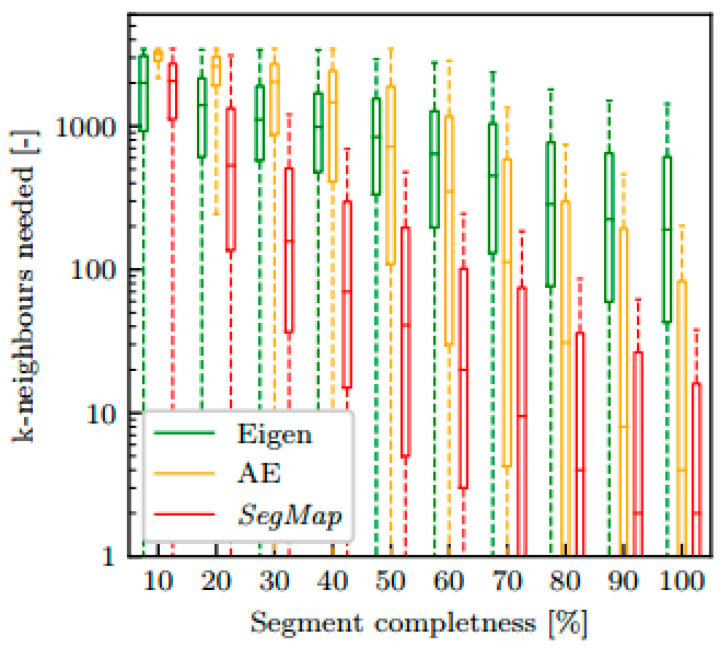
Impact of segment growth on descriptor relevance for localization [93].

The integration of semantic segmentation furnished robust cues to facilitate loop closure under diverse conditions by emphasizing stable background structures [91,92]. Semantic maps filtered out variable foreground objects and offered invariant scene representations for reliable matching against stored map data. Noteworthy works that leveraged semantic segmentation for enhanced loop closure detection included:

SegMap [93]: SegMap boosted loop closure detection performance by amalgamating semantic information with maps. This system adeptly identified and flagged loop closures to augment localization accuracy. Figure 8 presents how quickly descriptors extracted from incrementally grown segments contained relevant information that could be used for localization. The *x*-axis represents the growing status of a segment until all its measurements have been accumulated (here termed complete). The logarithmically scaled *y*-axis represents the number of neighbors in the target map that needed to be taken into account to include the correct target segment (lower values indicated better performance). The SegMap descriptor offered one order of magnitude better retrieval performance for over 40% of the growing process.

SIIS-SLAM [91,94]: SIIS-SLAM was an enhanced system derived from ORB-SLAM3 that incorporated semantic segmentation to enhance loop closure detection performance. This system could identify and track semantic targets, improving the effectiveness of SLAM. Additionally, the absolute locus RMSE was evaluated using a publicly available dataset, demonstrating superior performance compared to both the original ORB-SLAM3 and DynaSLAM results. According to the experimental results, the method outlined in this paper was found to be more suitable for indoor environments.

**Figure 9:**
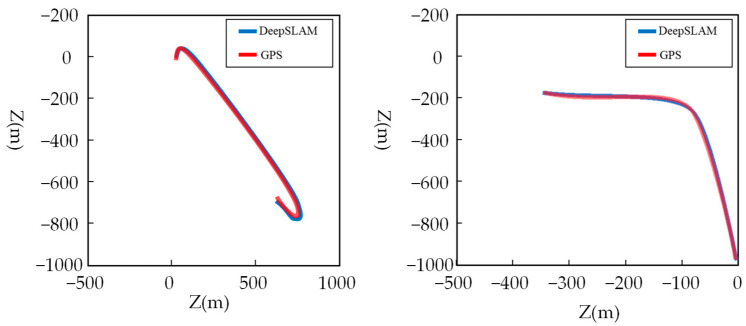
Testing on dataset using a low-cost ZED camera.

DeepSLAM [95]: DeepSLAM was a VSLAM system driven by deep learning, utilizing CNNs to process semantic segmentation data and integrating it into loop closure detection. This approach enhanced the accuracy and robustness of loop closure detection. Under typical weather conditions, the trajectory results obtained by DeepSLAM closely aligned with those from GPS/INS, as shown in Figure 9. However, experimental findings revealed that when faced with challenges including rain, nighttime conditions, and white balance variations, traditional LSD-SLAM and ORB-SLAM exhibited minimal efficacy, whereas DeepSLAM effectively leveraged prior knowledge acquired through training to perform well. DeepSLAM operated as a form of supervised learning.

These methods demonstrated enhanced loop closure detection capabilities compared to traditional approaches relying on manually crafted features including SIFT or ORB, especially in demanding perceptual scenarios. Semantic segmentation provided a robust high-level comprehension of the surroundings, enabling drift-free relocalization within VSLAM systems.

### 4.3. Environment Mapping

The map representation constituted another crucial component in VSLAM systems. Traditional maps typically consisted of geometric representations that primarily encoded low-level metric or topological information [95,96]. In contrast, semantic maps, which incorporated high-level context, provided a more comprehensive understanding of the environment, thereby enhancing intelligent interactions and decision-making capabilities.

**Figure 10:**
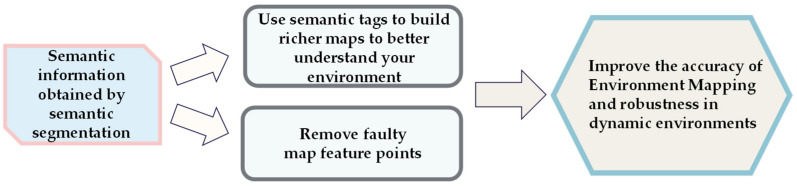
Semantic segmentation model improving the principle of environment mapping.

Semantic segmentation facilitated the enhancement of traditional maps by integrating pixel-level semantic labels to differentiate between various classes including walls, furniture, objects, and people [71]. Figure 10 illustrates the process of enhancing environment mapping with semantic segmentation models. This approach enabled unambiguous representations of environments as opposed to solely depicting surfaces or geometric primitives [97]. Noteworthy VSLAM systems that integrated semantics for improved mapping included the following.

Semantic Fusion SLAM [98]: Semantic Fusion integrated semantic segmentation from various perspectives with maps generated by Elastic Fusion. This approach enabled the creation of an effective semantic 3D map and enhanced the precision of single-frame semantic annotation. It established long-term dense correspondence between frames in indoor RGB-D videos, even during complex scanning trajectories. These correspondences facilitated the probabilistic fusion of semantic predictions from multiple viewpoints using CNNs into a map. This not only yielded a valuable semantic 3D map but also demonstrated, using the NYUv2 dataset, that merging multiple predictions led to an enhancement in 2D semantic labeling compared to baseline single-frame predictions.

**Figure 11:**
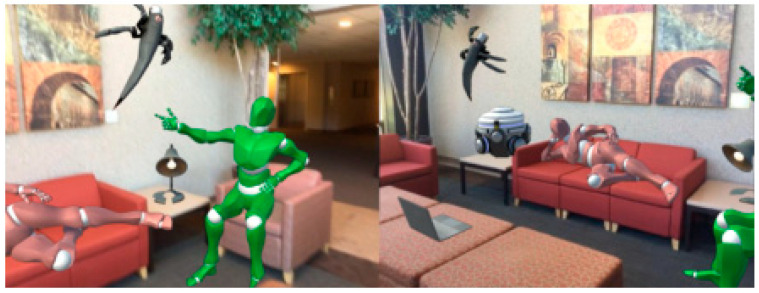
An example of AR application using a 3D panoptic map generated by the Panoptic Fusion system [99].

Panoptic Fusion [99]: Panoptic Fusion combined semantic segmentation and instance segmentation to generate 3D panoptic maps labeling each object instance and background classes. Additionally, researchers constructed a fully connected CRF model with respect to panoptic labels and performed online inference using a novel unary potential approximation and map division strategy, further improving recognition performance. This not only provided an effective semantic 3D map but also enhanced the accuracy of single-frame semantic annotation. Effective validation was achieved in AR scenarios, as shown in Figure 11.

LIO-SAM [100]: LIO-SAM was a VSLAM system based on semantic segmentation. It employed a semantic segmentation model to distinguish various objects and landmarks within the environment, consequently improving the precision of localization and map generation. For example, SAC-SLAM could identify buildings, roads, trees, and traffic signs, leading to the creation of more informative maps.

**Figure 12:**
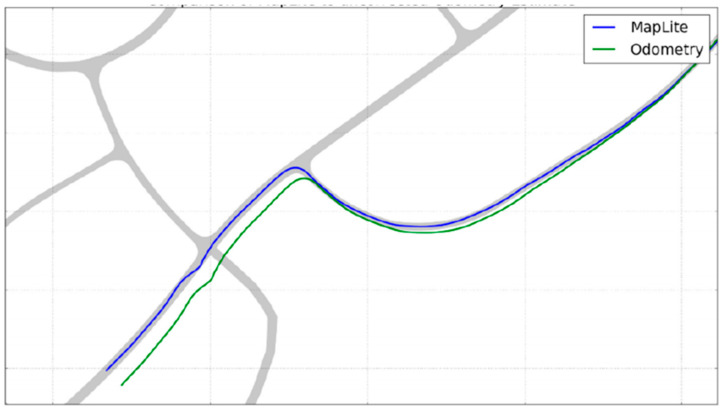
A comparison between the trajectory autonomously driven by MapLite (blue) with the path estimated by odometry (green). Note: the shaded area is the ground truth road surface [75].

MapLite [75]: MapLite integrated semantic pixel labels predicted by ERFNet into pose graph optimization for lifelong semantic mapping. These approaches yielded semantic maps with more detailed representations of environments by integrating class-level and instance-level semantic segmentations. This enhanced the system’s capability for scene comprehension, facilitating intelligent interactions and behaviors. The researchers conducted experiments on real-world roads, demonstrating that MapLite outperformed traditional visual odometry methods in trajectory estimation accuracy, as shown in Figure 12.

### 4.4. Model Action Mechanism

**Figure 13:**
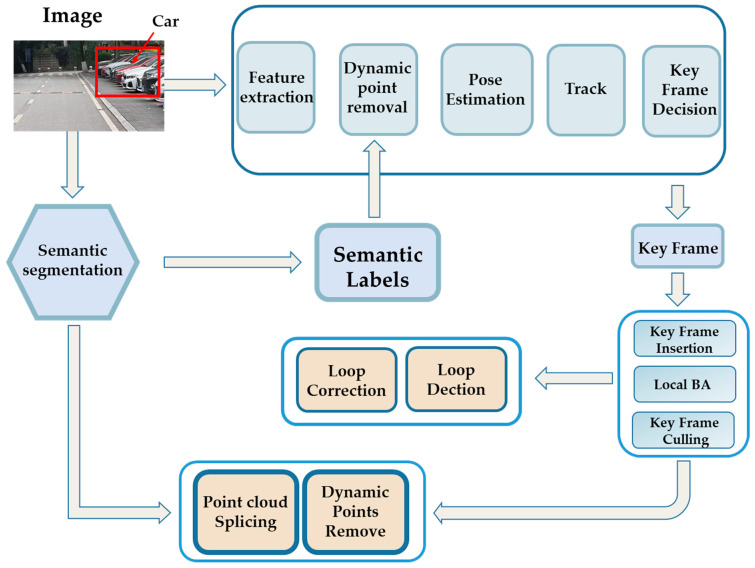
The mechanism of the semantic segmentation model in VSLAM.

The traditional VSLAM algorithm assumed that the objects in the environment were static or exhibited low motion. However, the presence of dynamic objects, including cars, could introduce erroneous data to the VSLAM system, thereby reducing its accuracy and robustness. As the camera might not accurately capture dynamic object data, some of the aforementioned examples utilized the semantic segmentation algorithm to filter out dynamic areas in the image. Subsequently, they used static environmental points to calculate the camera’s position nearby and construct a map containing semantic information. Figure 13 shows a classic structure. Although the complete elimination of the influence of dynamic objects might not have been achievable, the robustness of the system was significantly enhanced.

Through our analysis, the semantic segmentation model can provide a more accurate understanding of the environment, thereby improving the positioning accuracy of VSLAM, and by identifying semantic objects in the environment, VSLAM can better estimate the camera pose. By being able to recognize objects in the environment, semantic segmentation models help VSLAM perform smarter tasks in complex environments, such as avoiding obstacles or choosing the best path. Table 3 summarizes some usage scenarios and characteristics of VSLAM combined with the semantic segmentation model.

### 4.5. Experimental Comparison

The previous research suggested that a semantic segmentation model could help VSLAM mitigate the impact of dynamic objects on localization and mapping. Subsequently, the team conducted comparative experiments on the KITTI dataset, with the experimental results outlined in Table 4. The findings illustrated that VSLAM integrated with a semantic segmentation model demonstrated superior performance in dynamic environments.

### 4.6. Discussion

The integration of semantic segmentation proved to be a valuable strategy for enhancing traditional geometry-based VSLAM systems. By providing pixel-level semantic understanding of environments, semantic segmentation enabled the differentiation between stable background structures and dynamic foreground objects [101]. This enhancement benefited crucial components of the VSLAM pipeline, including visual odometry, loop closure detection, and mapping.

In the realm of visual odometry, semantic segmentation facilitated the identification and exclusion of features associated with movable objects during ego-motion estimation between frames. Compared with traditional methods, some VSLAM systems showed improved accuracy and robustness in dynamic environments. For loop closure detection, semantics assisted in retrieving stable structural scene elements for reliable relocalization despite variations in viewpoint and lighting conditions. In terms of mapping, semantic labels complemented geometric representations with class-level understanding to construct more detailed contextual maps of environments.

However, challenges persisted in the integration of semantics into VSLAM systems. Semantic segmentation models heavily relied on large, annotated training datasets, which were still limited in many robotics domains. These models also needed to be lightweight and efficient for real-time inference on resource-constrained platforms [102]. Numerous research issues remained unresolved, including optimal network architectures, accelerated model deployment, automated data annotation, online adaptation, and seamless integration with various VSLAM components.

Overall, semantic segmentation unlocked novel perception capabilities that paved the way for next-generation VSLAM systems characterized by intelligence, context awareness, and resilience to environmental dynamics. This emerging field held immense potential and presented numerous open challenges for future exploration. Advanced semantic segmentation techniques would serve as fundamental building blocks for achieving reliable VSLAM in complex real-world scenarios.

## 5. Conclusions

This study provided a comprehensive overview of integrating semantic segmentation into VSLAM systems. Fundamental technologies for traditional VSLAM systems were initially introduced, and the basic principles of semantic segmentation, with a focus on deep learning-based approaches, were outlined. Subsequently, the applications of semantic segmentation in key components of the VSLAM pipeline were delineated, and representative solutions were compared. Finally, the challenges and future directions in this emerging field were discussed. The key conclusions can be drawn as follows:

(1) Semantic segmentation assisted in distinguishing between static and dynamic elements in complex environments, thereby enhancing VSLAM accuracy and robustness. However, issues including sensor noise, environmental variations, and algorithm constraints could lead to the accumulation of positioning errors over prolonged periods. Nevertheless, semantic segmentation provided only partial information. To gain a more comprehensive understanding of the environment, further integration of semantic segmentation results with other sensor data, including inertial measurement units, LiDAR, etc., was identified as a worthwhile research direction to further enhance the performance of VSLAM systems.

(2) Significant applications included the identification of movable objects for visual odometry, the retrieval of structural features for loop closure detection, and the incorporation of semantic labels for contextual mapping. However, due to the imbalanced distribution of pixels among different categories in real-world settings, semantic segmentation models could exhibit suboptimal performance on minority classes, potentially impacting mapping outcomes.

(3) Promising research directions for the future involved dynamically adjusting the weights of semantic segmentation models based on scene dynamics to better adapt to varying environments. Additionally, considering the time consistency of semantic information could reduce the positioning errors caused by discontinuity. For example, utilizing semantic consistency across frames could help address issues in closed-loop detection. Optimizing semantic segmentation algorithms for real-time VSLAM systems to reduce computational resource consumption emerged as a critical area of interest.

(4) In highly dynamic scenes, the positioning accuracy of semantic VSLAM was significantly improved. However, in environments with untextured areas or repetitive patterns, semantic VSLAM might not function effectively. In these cases, employing target detectors to identify potential moving objects and eliminate their regions could mitigate the impact of dynamic objects on pose estimation.

In summary, essential perception capabilities were provided by semantic segmentation, paving the way for next-generation intelligent VSLAM systems. With the continuous advancement of deep neural networks and accelerated computing, semantically enriched VSLAM was poised to unleash the full potential of autonomous robots and agents operating in real-world scenarios. It is obvious that valuable insights were offered by this review to steer future research endeavors in this domain.

## 6. Future Trends and Prospects

The integration of semantic segmentation into VSLAM systems represented an emerging field with numerous open challenges and exciting opportunities. Based on this review, several promising directions for future research are identified:

Data-Driven Approaches: The availability of larger, high-quality labeled datasets tailored to robotics domains could significantly advance semantic segmentation for VSLAM. Techniques including weakly supervised [103], semi-supervised [104], and unsupervised methods [105] like self-training could mitigate annotation requirements. Additionally, synthetic data generation and domain adaptation methods could enhance model generalization.

Lightweight Architectures: The development of compact and efficient model designs was essential to enable real-time semantic segmentation on embedded systems with limited computing resources. Techniques including network pruning [106], knowledge distillation, quantization, and other optimization methods could tailor complex models for efficient deployment.

Online Adaptation Online learning [107] and domain adaptation algorithms [108] could progressively enhance segmentation models by adapting to changing environments encountered during operation, thereby improving reliability for lifelong VSLAM.

Multimodal Fusion: The fusion of complementary modalities including RGB, depth, thermal, LiDAR, and radar [106] at both input and feature levels could enhance segmentation accuracy, robustness, and consistency, thereby benefiting various components of VSLAM.

Dynamic Reconstruction: The combination of semantics with geometry could enable precise reconstruction and tracking of dynamic objects and interaction hotspots, contributing to safer navigation and smarter decision-making [35].

System Integration: Deeper integration between semantic segmentation modules and other VSLAM components could lead to end-to-end joint optimization and enhanced overall performance [109].

By advancing these research directions, semantically enriched VSLAM systems could unlock smarter interactions, robust long-term autonomy, and reliable performance in unstructured dynamic environments. It was anticipated that semantics would become indispensable to all perception-driven robots and intelligent agents operating in the real world.

## Figures and Tables

**Table 1:** The advantages and disadvantages of some main regularization methods at present.

Method	Standard Discussion
Volumetric	Volume integrity: Consider the volume information in the map, including the volume of a building, room, or other object. This can be assessed by comparing the volume in the map to the volume in the actual scene.
	Volume consistency: Check that the volume of different areas in the map is consistent. If the volume in the map changes too much, it may indicate that the map is incomplete.
Surface	Surface integrity: Focus on surface information in the map, including walls, floors, and ceilings. A complete map should accurately capture the geometry of these surfaces.
	Surface consistency: Check that surfaces in different areas of the map are consistent. If the surface shape in the map is inconsistent, it may indicate that there is a problem with the map.
Semantic classifications	Semantic integrity: Consider the semantic information in the map, including the categories of objects (e.g., chairs, tables, doors, etc.). A complete map should be able to mark these objects correctly.
	Semantic consistency: Check whether semantic labels in different areas of the map are consistent. If the semantic labels in the map are inconsistent, it may indicate that the map is incomplete or contains errors.

**Table 2:** The main semantic segmentation model and its application and characteristics.

Model	Features	Application Scenarios
U-Net [79]	Simple, efficient, easy to build.	U-Net can classify image pixels into different categories, including lanes, stop lines, speed bumps, and obstacles.
Mask-RCNN [76]	Features: Powerful image-based instance level segmentation algorithm.	Mask-RCNN can segment instances of different semantic objects at the pixel level, which is suitable for dynamic environments.
Pyramid Scene Parsing Network [45]	Considering the context relationship matching problem, showing a good segmentation effect.	PSPNet performs well in complex environments and can extract semantic information efficiently.
Fully Convolutional Networks [80]	The traditional convolutional neural network is transformed into a full convolutional structure for pixel-level semantic segmentation.	FCN is widely used in semantic segmentation tasks, which can effectively extract semantic information from images.
ERFNet [78]	Real-time segmentation with low computational costs while maintaining high accuracy.	ERFNet is particularly suitable for scenarios that require real-time performance, including autonomous driving and lane detection.

**Table 3:** Some common VSLAM usage scenarios and features.

Model	Semantic Segmentation Model	Features	Application Scenario
MaskFusion [84]	Mask R-CNN	Object-level RGB-D SLAM system for dynamic environments. Run in real time, able to track multiple moving objects and perform dense reconstruction.	Autonomous driving, online positioning at the vehicle end.
VDO-SLAM [85]	FCN	Emphasis on dynamic object perception, without the need for an a priori model of the object. The motion estimation of rigid objects is realized by using semantic information.	Deployment in real-world applications involving highly dynamic and unstructured environments.
ORB-SLAM3 [86]	Mask R-CNN	Real-time calculation of camera position and generate sparse 3D reconstructed maps.	Mobile robots, mobile phones, drones.
SegMap [93]	Mask R-CNN	A pure static semantic octree map is constructed by using semantic information.	Construction, navigation.
Semantic Fusion SLAM [98]	Res-Net	Real-time: Systems typically need to operate in a real-time environment and therefore require high frame rates and low latency.	High-precision map construction for autonomous vehicles.

**Table 4:** Comparison of the ATE [m] RMSE.

Sequence	ORB-SLAM	DynaSLAM	SLAM-Net	Semantic- Assisted SLAM	ORB-SLAM3	SIIS-SLAM
KITTI 01	5.19	7.44	5.06	5.12	4.76	7.23
KITTI 02	23.45	26.53	22.36	22.63	19.68	23.36
KITTI 03	1.49	1.79	1.43	1.53	1.55	1.78
KITTI 04	1.58	0.99	1.56	1.49	1.45	0.93
KITTI 05	4.79	4.53	4.66	4.23	3.99	4.55
KITTI 06	13.01	14.79	12.36	11.36	10.77	12.88
KITTI 07	2.30	2.26	2.12	2.19	2.08	2.26
KITTI 08	47.69	41.23	46.23	45.26	43.16	39.31
KITTI 09	6.53	3.22	5.99	5.36	4.23	2.96

## Data Availability

Not applicable.
